# NNMT Is an Immune-Related Prognostic Biomarker That Modulates the Tumor Microenvironment in Pan-Cancer

**DOI:** 10.1155/2023/9226712

**Published:** 2023-02-09

**Authors:** Wenxiu Liu, Meng Zhu, Xiaoming Li, Limian Er, Shengmian Li

**Affiliations:** ^1^Department of Gastroenterology and Hepatology, The Fourth Hospital of Hebei Medical University, Shijiazhuang, Hebei, China; ^2^Hainan Yilai Telemedicine Center, Hainan, China; ^3^Department of Endoscope room, The Fourth Hospital of Hebei Medical University, Shijiazhuang, Hebei, China

## Abstract

Emerging evidence has revealed the significant roles of nicotinamide n-methyltransferase (NNMT) in cancer initiation, development, and progression; however, a pan-cancer analysis of NNMT has not been conducted. In this study, we first thoroughly investigated the expression and prognostic significance of NNMT and the relationship between NNMT and the tumor microenvironment using bioinformatic analysis. NNMT was significantly increased and associated with poor prognosis in many common cancers. NNMT expression correlated with the infiltration levels of cancer-associated fibroblasts and macrophages in pan-cancer. Function enrichment analysis discovered that NNMT related to cancer-promoting and immune pathways in various common cancers, such as colon adenocarcinoma, head and neck squamous cell carcinoma, ovarian serous cystadenocarcinoma, and stomach adenocarcinoma. NNMT expression was positively correlated with tumor-associated macrophages (TAMs), especially M2-like TAMs. The results suggest that NNMT might be a new biomarker for immune infiltration and poor prognosis in cancers, providing new direction on therapeutics of cancers.

## 1. Introduction

Cancer ranks as a leading cause of death, and the burden of cancer incidence and mortality is growing rapidly worldwide [1]. The Global Cancer Statistics 2020 estimates indicated that there were 19.3 million new cancer cases and almost 10 million cancer-related deaths in 2020 [2]. The etiology and tumorigenic process are extremely complicated and occur in concert with alterations in the surrounding stroma. The tumor microenvironment (TME) is an integral part of cancer, comprising various cell types (stromal cells, fibroblasts, endothelial cells, immune cells, etc.) and extracellular components (extracellular matrix, growth factors, hormones, cytokines, etc.) [3]. The TME not only plays a pivotal role during tumor progression and metastasis but also has profound effects on the therapeutic efficacy [4]. Innate or adaptive immune cells in TME have both anticancer and protumor activities, and accumulation of immune infiltrating cells in TME is involved in tumor development [5]. Former study has proved that oncogenes can rebuild TME and promote cancer development by directly or indirectly influencing immune cells or stromal cells. As an alternative to classic antitumor treatments, immunotherapy has verified efficacy in many different cancer types and been built up to reactivate adaptive and innate immune systems, which targets interactions between immune cells and cancer cells [6]. For example, programmed death ligand-1 (PD-L1) and programmed death-1 (PD-1) were found to have promising anticancer effects on some cancers. Unfortunately, present immunotherapies have been effective only in a few cancer patients with specific type but not in others. In consequence, it is urgent to identify new oncogenes that play key roles in TME and validate the new immune-related therapeutic targets for cancers.

Nicotinamide N-methyltransferase (NNMT) is a metabolic enzyme that catalyzes the methylation of nicotinamide using the universal methyl donor S-adenosyl methionine, directly linking one-carbon metabolism with the balance between the cell's methylation level and nicotinamide adenine dinucleotide levels [7]. Recent research showed that NNMT promoted tumorigenesis in several ways, including facilitating cancer cell proliferation and migration, inhibiting autophagy, and regulating the differentiation of cancer-associated fibroblasts (CAFs) in the stroma [8–11].

However, most studies on the function of NNMT in cancers have been limited to a specific type of cancer. Its biological effects are not entirely known, and the role of NNMT in TME remains unclear. Whether NNMT can play a role in the pathogenesis of different tumors through certain common molecular mechanisms remains to be answered. Therefore, it is particularly important to conduct an in-depth examination of the regulatory functions of NNMT in a pan-cancer dataset to provide new directions and strategies for the clinical treatment of cancer.

The present study first consistently characterized the prevalence and prognostic value of NNMT expression in pan-cancer. Meanwhile, the molecular mechanism of NNMT in the cancer occurrence and progression and its function in TME and immune cell infiltration was also discussed. Generally, our first systematically pan-cancer analysis showed that NNMT expression was closely related with cancer patient prognosis and immune cell infiltration. NNMT played an oncogenic role in pan-cancer, and high NNMT expression had a bad effect on the survival time of cancer patients. NNMT expression remarkably correlated with the infiltration levels of CAFs and macrophages. These findings implied that NNMT influenced the clinical prognosis of cancer patients, probably by means of its interaction with tumor-infiltrating immune cells.

## 2. Materials and Methods

### 2.1. Analysis of NNMT Expression in Various Types of Tumors

NNMT mRNA expression levels in different cancer types were compared with their matched adjacent normal tissues by using web-based Oncomine analysis tools. Oncomine (https://www.oncomine.org/) is a major cancer microarray repository and web-based data mining platform [12], which contains 715 datasets and 86,733 samples. The thresholds were set at a *P* value of 0.001 and a fold change of 1.5 in our experiment. The NNMT expression profile and the abundance of immune infiltrates in pan-cancer were analyzed using the TIMER database (https://cistrome.shinyapps.io/timer). The gene expression levels are represented as log2 (TPM (transcripts per million) +1) values. The NNMT protein expression levels were analyzed using the Clinical Proteomic Tumor Analysis Consortium (CPTAC) database from UALCAN (http://ualcan.path.uab.edu/analysis).

Next, we used the “Expression analysis-Box Plots” module of the Gene Expression Profiling Interactive Analysis2 (GEPIA2) web server (http://gepia2.cancer-pku.cn/) to obtain the violin plots of NNMT expression at different pathological stages (stages I, II, III, and IV) of all cancers in The Cancer Genome Atlas (TCGA) [13]. The log2 TPM transformed expression data were applied to the violin plots.

### 2.2. Analysis of the Correlation between NNMT Expression and Patient Survival

The correlation between NNMT expression and survival in pan-cancer was analyzed using the Kaplan-Meier plotter (https://kmplot.com/analysis/) and GEPIA (http://gepia.cancer-pku.cn/). The Kaplan-Meier plotter is a powerful online tool that can be used to assess the effect of 54,675 genes on survival using 10,461 cancer samples in 21 cancer types [14]. We analyzed the relationship of NNMT expression with overall survival (OS) and relapse-free survival (RFS) in each available cancer type using the Kaplan-Meier plotter. The hazard ratio (HR) with 95% confidence intervals (CI) and logrank *P* value were computed. GEPIA is an interactive online platform with tumor sample information from TCGA and normal sample information from TCGA and Genotype-Tissue Expression project [15]. We explored the effect of NNMT expression on OS and disease-free survival (DFS) in 34 cancer types using GEPIA. A cut-off value of 50% was used as the expression threshold to distinguish the high expression and low expression cohorts. The logrank test was used in the hypothesis test, and the survival plots were obtained through the “Survival Analysis” module of GEPIA.

### 2.3. Correlation of NNMT Expression with the Tumor Microenvironment in Pan-Cancers

Using TCGA NNMT expression data, the stromal and immune cell scores were calculated after applying the ESTIMATE algorithm in R-package “estimate” and “limma” [16] for predicting the presence of infiltrating stromal/immune cells in pan-cancer tissues. The correlation analysis of NNMT expression with the tumor microenvironment (TME) and immune cell infiltration was pursued using the R-package “ggplot2,” “ggpubr,” and “ggExtra” (*P* < 0.001 as a cut-off value).

We used the “Immune-Gene” module of the TIMER2 web application to explore the association between NNMT expression and immune infiltrates across all TCGA tumors. The TIMER, CIBERSORT, CIBERSORT-ABS, QUANTISEQ, XCELL, MCPCOUNTER, and EPIC algorithms were applied for immune infiltration estimations. The *P* values and partial correlation values were obtained via the purity-adjusted Spearman rank correlation test. The data were visualized as a heatmap and a scatter plot.

Next, we confirmed the association between NNMT expression and immune infiltrates in pan-cancer using Immune Cell Abundance Identifier (ImmuCellAI; http://bioinfo.life.hust.edu.cn/ImmuCellAI). The ImmuCellAI is a tool to estimate the abundance of 24 immune cells from a gene expression dataset, including RNA sequencing and microarray data, wherein the 24 immune cells comprise 18 T cell subtypes and 6 other immune cells: B cell, dendritic cells (DCs), natural killer (NK) cell, neutrophils, monocytes, and macrophages. Cancer samples were divided into two groups according to the median NNMT expression (high versus low level), and their immune cell infiltration levels were compared.

The correlations between NNMT expression and gene markers of tumor-associated macrophages (TAMs) as well as M1 and M2 macrophages in colon adenocarcinoma (COAD), head and neck squamous cell carcinoma (HNSC), ovarian serous cystadenocarcinoma (OV), and stomach adenocarcinoma (STAD) were analyzed in the TIMER database.

### 2.4. Correlation and Enrichment Analyses

The Pearson correlation analysis of NNMT mRNA and other mRNAs was performed in the COAD, HNSC, OV, and STAD using TCGA data. The 300 genes most positively associated with NNMT were selected for an enrichment analysis to determine the function of NNMT. Gene set enrichment analysis (GSEA) was performed using the gseGO, gseKEGG, and gsePathway functions in the clusterProfiler R software package R with the following parameters: nPerm = 1,000, minGSSize = 10, maxGSSize = 1,000, and *P* < 0.05 as a cut-off value.

### 2.5. Statistical Analysis

Survival curves were generated by the Kaplan-Meier plots and GEPIA. The results generated in Oncomine were displayed with *P* values, fold changes, and ranks. The results of the GEPIA and Kaplan-Meier plots were displayed with HR and Por Cox *P* values from a logrank test. The correlations between NNMT expression and abundance scores of stromal/immune cells were evaluated by Spearman's correlation. The strength of the correlation was determined using the following guide for the absolute value: very weak, in between 0.00 and 0.19; weak, in between 0.20 and 0.39; moderate, in between 0.40 and 0.59; strong, in between 0.60 and 0.79; and very strong, in between 0.80 and 1.0. The results with *P* < 0.05 were considered as statistically significant.

## 3. Results

### 3.1. NNMT Expression in Different Cancer Types

**Figure 1:**
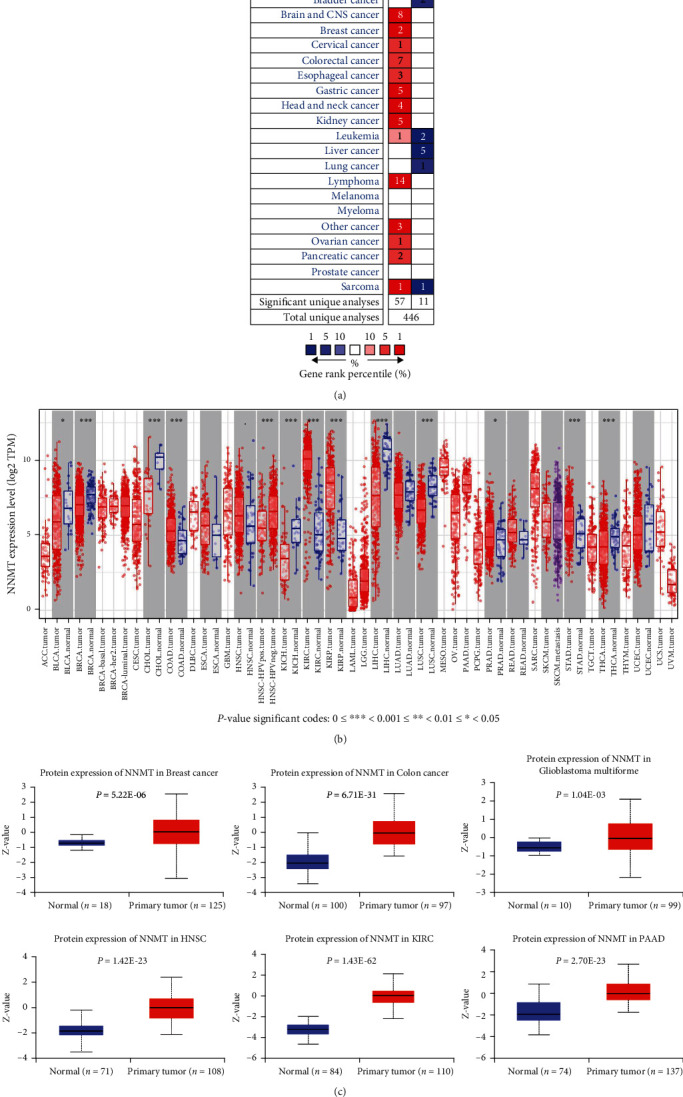
The NNMT mRNA expression levels in different human cancer types. (a) Increased or decreased expression of NNMT compared with normal tissues across different cancer types in the Oncomine database. (b) The NNMT mRNA expression levels in different cancer types from TCGA database in TIMER (^∗^*P* < 0.05, ^∗∗^*P* < 0.01, and ^∗∗∗^*P* < 0.001). (c) The NNMT protein expression levels in normal tissues and primary tissues of the breast cancer, colon cancer, glioblastoma multiforme, HNSC, KIRC, and PAAD were examined using the CPTAC dataset. CPTAC: Clinical Proteomic Tumor Analysis Consortium; HNSC: head and neck squamous cell carcinoma; KIRC: kidney renal clear cell carcinoma; NNMT: nicotinamide N-methyltransferase; PAAD: pancreatic adenocarcinoma.

First, we evaluated the NNMT mRNA levels in diverse cancers and their matched adjacent normal tissues over a cancer-wide range in Oncomine and TIMER databases. Compared with the normal tissues, in Oncomine database, the results revealed higher expression of NNMT in brain and central nervous system (CNS), breast, cervix, colon, esophagus, stomach, head and neck, kidney, lymphatic system, ovary, and pancreas cancers (Figure 1(a)). In contrast, decreased expression of NNMT was found in bladder, liver, and lung cancers.

To further verify the expression levels of NNMT in cancerous and normal tissues across all TCGA tumors, we profiled and compared them in TIMER platform. Specifically, NNMT expression levels were significantly elevated in colon adenocarcinoma (COAD), head and neck squamous cell carcinoma (HNSC), kidney renal clear cell carcinoma (KIRC), kidney renal papillary cell carcinoma (KIRP), and stomach adenocarcinoma (STAD); these results were consistent with those in the Oncomine database. In contrast, decreased expression of NNMT was found in bladder urothelial carcinoma (BLCA), breast invasive carcinoma (BRCA), cholangiocarcinoma (CHOL), kidney chromophobe (KICH), liver hepatocellular carcinoma (LIHC), lung squamous cell carcinoma (LUSC), and thyroid carcinoma (THCA) (Figure 1(b)).

Further comparison of NNMT protein expression according to the CPTAC database from UALCAN demonstrated that NNMT protein expression levels were significantly higher in advanced tumor tissues than in normal tissues; this was observed in breast cancer, colon cancer, glioblastoma multiforme, HNSC, KIRC, and pancreatic adenocarcinoma (PAAD) (Figure 1(c), all *P* < 0.001).

**Figure 2:**
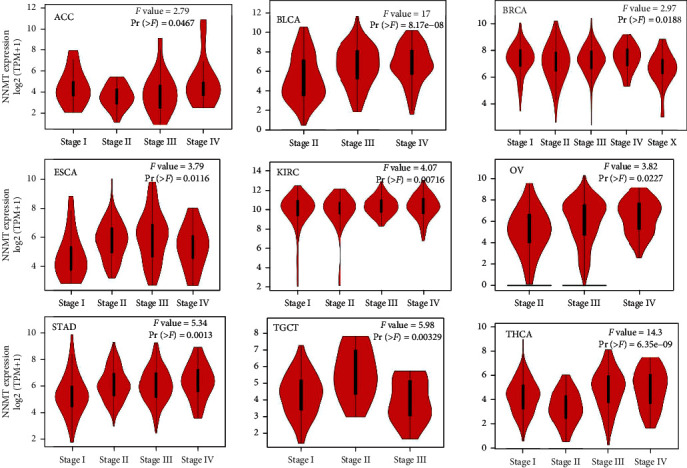
Association of NNMT expression level with different pathological stages. The expression levels of the NNMT were analyzed by the pathological stages (stages I, II, III, and IV) of ACC, BLCA, BRCA, ESCA, KIRC, OV, STAD, TGCT, and THCA from TCGA database in GEPIA. Log2 (TPM+1) was applied for log-scale. ACC: adrenocortical carcinoma; BLCA: bladder urothelial carcinoma; BRCA: breast invasive carcinoma; ESCA: esophageal carcinoma; GEPIA: Gene Expression Profiling Interactive Analysis; KIRC, kidney renal clear cell carcinoma; NNMT, nicotinamide N-methyltransferase; OV, ovarian serous cystadenocarcinoma; STAD, stomach adenocarcinoma; TGCT, testicular germ cell tumors; THCA, thyroid carcinoma; TPM, transcripts per million.

We also used the “Pathological Stage Plot” module of GEPIA2 to observe the correlation between NNMT expression and the pathological stages of cancers in all TCGA tumors. NNMT expression was significantly associated with the TNM stage in adrenocortical carcinoma (ACC), BLCA, BRCA, esophageal carcinoma (ESCA), KIRC, ovarian serous cystadenocarcinoma (OV), STAD, testicular germ cell tumors (TGCT), and THCA. As shown in Figure 2, NNMT expression level was significantly different from stage I to IV in ACC (*F* value = 2.79, *Pr* = 0.0467), BLCA (*F* value = 17, *Pr* = 8.17*E* − 08), BRCA (*F* value = 2.97, *Pr* = 0.0188), ESCA (*F* value = 3.79, *Pr* = 0.0116), KIRC (*F* value = 4.07, *Pr* = 0.00716), OV (*F* value = 3.82, *Pr* = 0.0227), STAD (*F* value = 5.34, *Pr* = 0.0013), TGCT (*F* value = 5.98, *Pr* = 0.00329), and THCA (*F* value = 14.3, *Pr* = 6.35*E* − 09). It seemed that NNMT expression was higher in the stage III-IV but lower in stage I-II patients in BLCA, OV, STAD, and THCA.

### 3.2. Prognostic Potential of NNMT in Cancers

**Figure 3:**
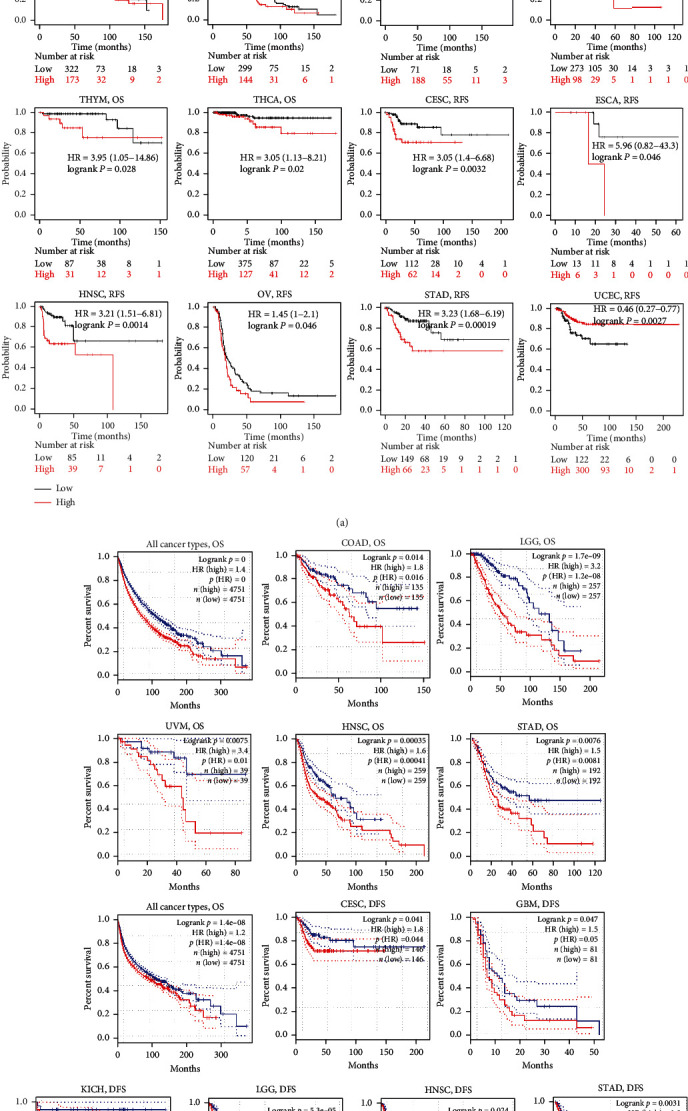
The Kaplan-Meier survival curves of NNMT expression in different cancer types. (a). Relationship between NNMT expression and the OS or RFS of cancer patients in the Kaplan-Meier Plotter. (b). Relationship between NNMT expression and the OS or DFS of cancer patients in the GEPIA. DFS, disease-free survival; GEPIA, Gene Expression Profiling Interactive Analysis; NNMT: nicotinamide N-methyltransferase; OS: overall survival; RFS: relapse-free survival.

Next, we analyzed the prognostic value of NNMT expression in pan-cancers using the Kaplan-Meier plotter and GEPIA data. The results in the Kaplan-Meier plotter database showed that 10 out of 21 cancer types displayed poorer prognosis, including bladder carcinoma (OS : HR = 1.53, 95%CI = 1.05 to 2.22, logrank *P* = 0.026), cervical squamous cell carcinoma and endocervical adenocarcinoma (CESC) (OS : HR = 1.71, 95%CI = 1.07 to 2.74, logrank *P* = 0.023; RFS : HR = 3.05, 95%CI = 1.4 to 6.68, logrank *P* = 0.0032), HNSC (OS : HR = 1.66, 95%CI = 1.27 to 2.19, logrank *P* = 0.00021; RFS : HR = 3.21, 95%CI = 1.51 to 6.81, logrank *P* = 0.0014), KIRC (OS : HR = 1.69, 95%CI = 1.23 to 2.31, logrank *P* = 0.0011), LUSC (OS : HR = 1.4, 95%CI = 1.07 to 1.85, logrank *P* = 0.016), OV (OS : HR = 1.35, 95%CI = 1.04 to 1.77, logrank *P* = 0.025; RFS : HR = 1.45, 95%CI = 1.0 to 2.1, logrank *P* = 0.046), STAD (OS : HR = 1.92, 95%CI = 1.37 to 2.69, logrank *P* = 0.00012; RFS : HR = 3.23, 95%CI = 1.68 to 6.19, logrank *P* = 0.00019), thymoma (THYM) (OS : HR = 3.95, 95%CI = 1.05 to 14.86, logrank *P* = 0.028), THCA (OS : HR = 3.05, 95%CI = 1.13 to 8.21, logrank *P* = 0.02), and ESCA (RFS : HR = 5.96, 95%CI = 0.82 to 43.3, logrank *P* = 0.046) (Figure 3(a)). Only in sarcoma (OS : HR = 0.63, 95%CI = 0.42 to 0.96, logrank *P* = 0.03) and uterine corpus endometrial carcinoma (UCEC) (RFS : HR = 0.46, 95%CI = 0.27 to 0.77, logrank *P* = 0.0027), high NNMT expression was associated with better prognosis (Figure 3(a)).

To further clarify the function of NNMT in pan-cancer, the GEPIA database, which can provide more cancer types, was used. Equally, higher NNMT mRNA levels revealed a worse prognostic prediction in all cancer types (OS : HR (high) = 1.4, logrank *P* = 0; disease − free survival (DFS): HR(high) = 1.2, logrank *P* = 1.4*E* − 08). NNMT high expression had a poorer prognosis in certain cancer types from GEPIA database, including COAD (OS : HR (high) = 1.8, logrank *P* = 0.014), HNSC (OS : HR (high) = 1.6, logrank *P* = 0.00035; DFS : HR (high) = 1.5, logrank *P* = 0.024), brain lower-grade glioma (LGG) (OS : HR (high) = 3.2, logrank *P* = 1.7*E* − 09; DFS : HR (high) = 1.9, logrank *P* = 5.3*E* − 05), STAD (OS : HR (high) = 1.5, logrank *P* = 0.0076; DFS : HR (high) = 1.8, logrank *P* = 0.0031), uveal melanoma (UVM) (OS : HR (high) = 3.4, logrank *P* = 0.0075), CESC (DFS : HR (high) = 1.8, logrank *P* = 0.041), glioblastoma multiforme (GBM) (DFS : HR (high) = 1.5, logrank *P* = 0.047), and KICH (DFS : HR (high) = 4.4, logrank *P* = 0.041) (Figure 3(b)). These results were consistent with the findings in the Kaplan-Meier plotter database.

Taking together, these integrated analyses revealed that NNMT played a detrimental role in cancer development and had prognostic value in pan-cancer.

### 3.3. Correlation between NNMT Expression and Tumor Microenvironment (TME) in Pan-Cancer

**Figure 4:**
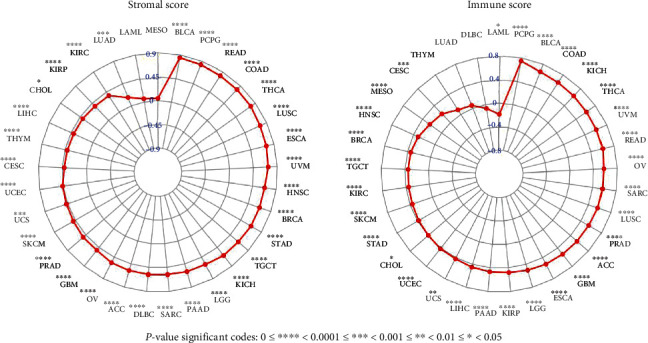
Correlation of NNMT gene expression with the stromal and immune scores in different cancers. (a) Correlation of NNMT gene expression with the stromal scores in pan-cancer. (b) Correlation of NNMT gene expression with the immune scores in pan-cancer. NNMT: nicotinamide N-methyltransferase.

The TME is essential in stimulating heterogeneity among cancer cells, thus leading to cancer cell progression and metastasis [17]. Since our findings validated the prognostic role of NNMT in pan-cancer, it would be highly appropriate to further explore the relationship between TME and NNMT expressions in different types of cancers. We used the ESTIMATE algorithm to calculate the stromal and immune cell scores in pan-cancer. The results showed that NNMT expression had significant correlations with stromal scores in 30 types of cancer and significant correlations with immune scores in 29 types of cancers. As shown in Figure 4(a) and Table S1, NNMT expression had very strong correlation with stromal scores in BLCA, PCPG, READ, and COAD (all *r* > 0.8, *P* < 0.0001); had strong correlation with stromal scores in THCA, LUSC, ESCA, UVM, HNSC, BRCA, STAD, TGCT, KICH, LGG, and PAAD (all *r* > 0.6, *P* < 0.0001); and had moderate correlation with stromal scores in SARC, DLBC, ACC, OV, GBM, PRAD, SKCM, UCS, UCEC, CESC, and THYM (all *r* > 0.4, *P* < 0.001). NNMT expression had strong correlation with immune scores in PCPG, BLCA, COAD, KICH, THCA, UVM, and READ (all *r* > 0.6, *P* < 0.0001) and had moderate correlation with immune scores in OV, SARC, LUSC, PRAD, ACC, GBM, ESCA, LGG, KIRP, PAAD, LIHC, UCS, and UCEC (all *r* > 0.4, *P* < 0.01) (Figure 4(b) and Table S1). This results would particularly important for COAD, HNSC, OV, and STAD since NNMT expression was increased and associated with poor prognosis in these types of cancer (according to the Kaplan-Meier or GEPIA analyses). NNMT expression had a significantly positive correlation to both stromal and immune scores in these types of cancer (Table S1 and Figure S1). These results indicated that the infiltration of stromal or immune cells escalates accompanied with the increase of NNMT expression in COAD, HNSC, OV, and STAD.

### 3.4. Association between NNMT Expression and Immune Cell Infiltration in Pan-Cancer

Tumor-infiltrating immune cells in the TME play a key part in the initiation, progression, recurrence, and metastasis of cancers. Here, we utilized some or all algorithms, including TIMER, EPIC algorithms, QUANTISEQ, CIBERSORT or CIBERSORT-ABS, MCPCOUNTER, and XCELL, to investigate the relationship between NNMT gene expression and the infiltration level of different immune cells in diverse cancers.

**Figure 5:**
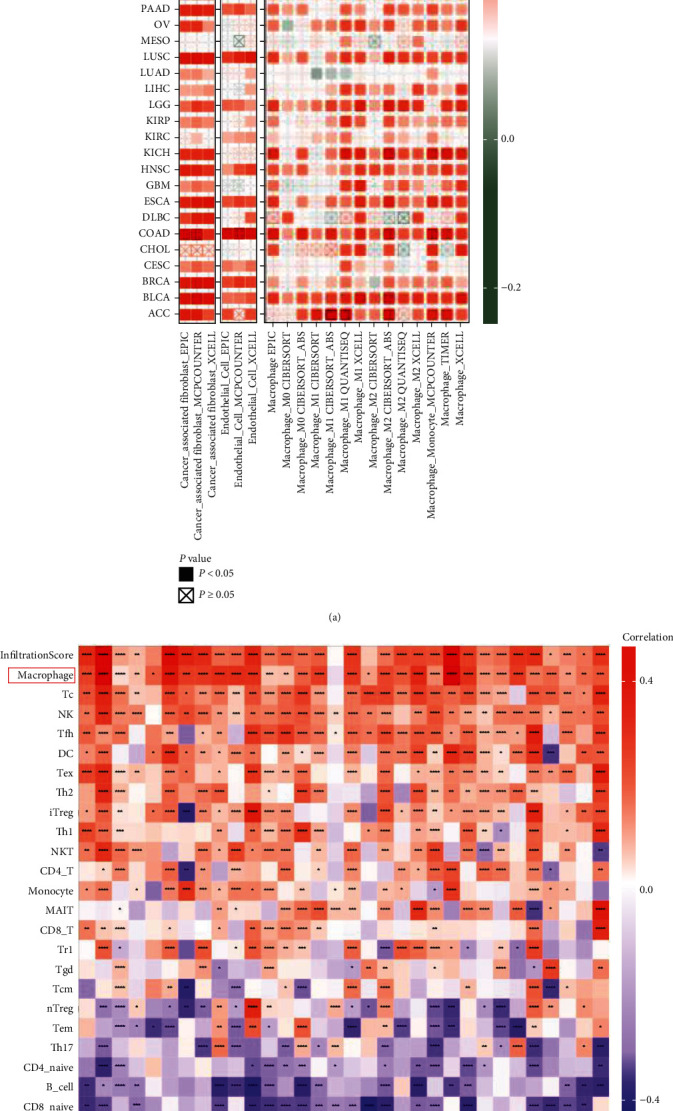
Correlation analysis between NNMT expression and immune cell infiltration. (a) NNMT expression significantly correlated with the infiltration levels of CAFs, endothelial cells, and macrophages in TCGA pan-cancer. (b) NNMT expression significantly correlated with the infiltration levels of various immune cells in the ImmuCellAI database. CAFs: cancer-associated fibroblasts; ImmuCellAI: Immune Cell Abundance Identifier; NNMT: nicotinamide N-methyltransferase; TCGA: The Cancer Genome Atlas.

The results showed that NNMT expression was closely related to the abundance of immune cells infiltrating: cancer-associated fibroblasts (CAFs) in 30 types of tumor, endothelial cells in 27 types of tumor, macrophages in 28 types of tumor, CD8^+^ T cells in 26 types of tumor, neutrophils in 25 types of tumor, CD4^+^ T cells in 22 types of tumor, natural killer (NK) cells in 20 types of tumor, and B cells in 11 types of tumor (Table S2). Compared with other immune cells, we observed a stronger correlation between NNMT expression and the infiltration of CAFs and macrophages in TCGA pan-cancer. The expression of NNMT had a positively strong correlations with infiltration levels of CAFs in COAD, READ, STAD, and BLCA (all *r* > 0.6, *P* < 0.0001), had a positively strong correlation with macrophages in READ (*r* = 0.63, *P* < 0.0001), and had moderate correlation with macrophages in UVM, BLCA, KICH, CHOL, LGG, COAD, PCPG, LIHC, SARC, GBM, UCS, DLBC, and LUSC (all *r* > 0.4, *P* < 0.001) (Figure 5(a) and Table S2).

To verify the association between NNMT expression and immune cell infiltration, we perform the correlation analysis in pan-cancer using ImmuCellAI. Consistent with the results in TCGA pan-cancer, macrophages were the most positively relevant immune cells with NNMT expression (Figure 5(b)). In addition, in cancers which NNMT expression levels were correlated with poor prognosis, such as COAD, HNSC, OV, and STAD, macrophage levels were significantly upregulated in the NNMT high expression group (Figure S2). In COAD, NNMT expression had significantly positive correlation with infiltrating levels of macrophages (*r* = 0.62, *P* = 1.35*E* − 31), dendritic cells (DCs) (*r* = 0.54, *P* = 2.09*E* − 22), T cells (*r* = 0.47, *P* = 4.80*E* − 17), induced regulatory T cells (iTregs) (*r* = 0.38, *P* = 5.66*E* − 11), T-helper 2 (Th2) cells (*r* = 0.38, *P* = 5.99*E* − 11), NK cells (*r* = 0.38, *P* = 7.03*E* − 11), and CD4^+^ T cells (*r* = 0.36, *P* = 6.73*E* − 10). For HNSC, the results also displayed that NNMT expression significantly related to infiltrating levels of macrophages (*r* = 0.53, *P* = 4.00*E* − 39), NKT cells (*r* = 0.46, *P* = 5.94*E* − 28), monocyte (*r* = 0.31, *P* = 3.78*E* − 13), and DCs (*r* = 0.29, *P* = 2.34*E* − 11). As same as COAD and HNSC, the correlation between NNMT and infiltrating levels of immune cells in OV is as follows: macrophage (*r* = 0.45, *P* = 6.49*E* − 17), T cells (*r* = 0.46, *P* = 3.56*E* − 17), follicular helper T (Tfh) cells (*r* = 0.44, *P* = 2.75*E* − 16), iTregs (*r* = 0.43, *P* = 6.38*E* − 15), Th2 cells (*r* = 0.40, *P* = 4.37*E* − 13), and DCs (*r* = 0.32, *P* = 8.75*E* − 09). Moreover, NNMT expression was positively related to CD4^+^ T cells (*r* = 0.33, *P* = 1.20*E* − 11), NK cells (*r* = 0.31, *P* = 9.07*E* − 11), and macrophages (*r* = 0.29, *P* = 1.20*E* − 09) in STAD (Figure 5(b) and Table S3). These results reflected that NNMT might influence cancer patient survival by affecting immune cell infiltration in the TME.

### 3.5. NNMT Correlates with Polarization of M2 Macrophage

The results above showed that macrophages was the most positively relevant immune cells with NNMT expression in pan-cancer. Next, we analyzed the correlation of NNMT and the gene markers of macrophage subtypes including tumor-associated macrophages (TAMs) and M1 and M2 macrophages in COAD, HNSC, OV, and STAD. The results showed that gene markers of TAM such as CCL2, CD68, CD80, and IL10 had strong or moderate correlations with NNMT expression. M2 macrophage markers including CD163, VSIG4, MRC1, and MS4A4A also had strong or moderate correlations with NNMT expression. However, M1 macrophage markers including INOS, IRF5, and ARG2 had negatively or no apparent correlations with NNMT expression (Table 1). These results indicated that NNMT may regulate the differentiation of macrophages into TAMs by affecting the polarization of M2 macrophage, which contributes to tumorigenesis and development.

### 3.6. Functional Enrichment Analysis of NNMT in COAD, HNSC, OV, and STAD

**Figure 6:**
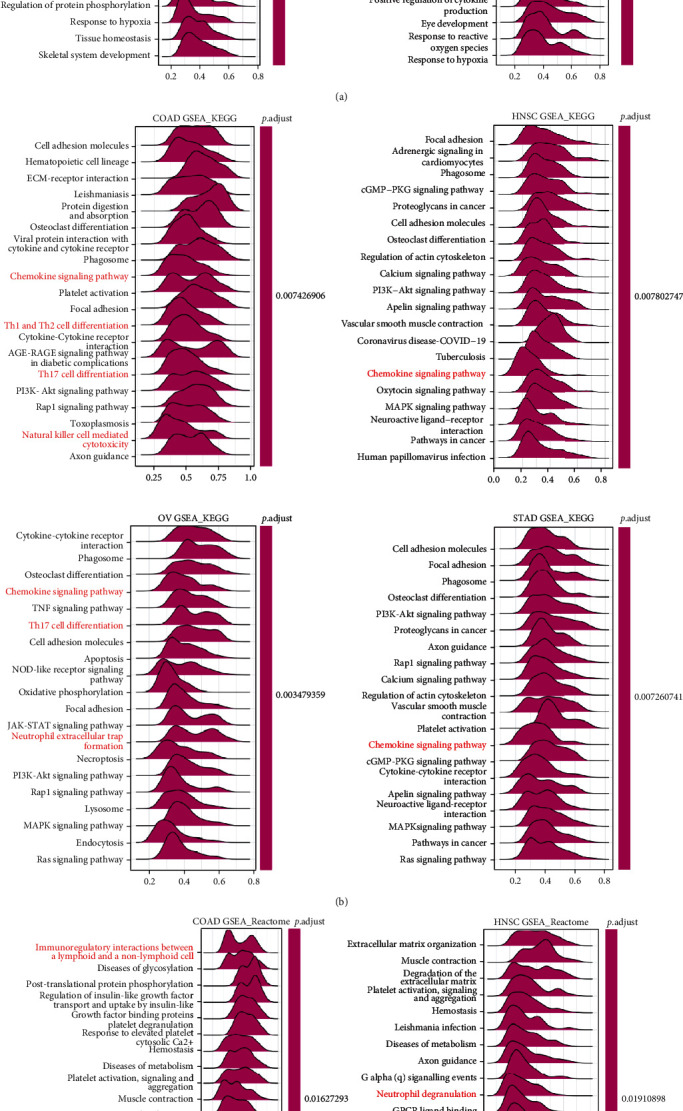
Merged enrichment plots for NNMT obtained from GSEA. (a–c) Merged plots of GSEA indicating the top 20 significant pathways associated with NNMT expression according to GO (a), KEGG (b), and Reactome analyses (c) in COAD, HNSC, OV, and STAD. Red color represents immune-related pathways. COAD: colon adenocarcinoma; GO: Gene Ontology; GSEA: Gene Set Enrichment Analysis; HNSC: head and neck squamous cell carcinoma; KEGG: Kyoto Encyclopedia of Genes and Genome; NNMT: nicotinamide N-methyltransferase; OV: ovarian serous cystadenocarcinoma; STAD: stomach adenocarcinoma.

We did Gene Set Enrichment Analysis (GSEA) of NNMT in COAD, HNSC, OV, and STAD to investigate the molecular mechanisms of NNMT in tumorigenesis and TME. Interestingly, we found the similar results in the selected cancers. NNMT was involved in a number of GO terms including MAPK cascade, regulation of cytokine production, and cytokine production in COAD, HNSC, OV, and STAD and angiogenesis in COAD, OV, and STAD (Figure 6(a)). GSEA results of KEGG analysis indicated that NNMT was associated with many cancer-promoting and immune-related pathways, such as the PI3K-Akt signaling pathways, cell adhesion molecules, and chemokine signaling pathways in COAD, HNSC, OV, and STAD; MAPK signaling pathways in HNSC, OV, and STAD; and cytokine-cytokine receptor interaction in COAD and OV (Figure 6(b)). The GSEA results for Reactome terms suggested that NNMT was positively regulated and provided several immune-related functions in COAD, HNSC, OV, and STAD. These activities included immunoregulatory interactions between a lymphoid and nonlymphoid cell, cytokine signalling in the immune system, adaptive and innate immune system, neutrophil degranulation, and interleukin-mediated signalling (Figure 6(c)). Generally, these results suggested that NNMT played a key role in carcinogenesis and tumor immune microenvironment.

## 4. Discussion

Nicotinamide N-methyltransferase (NNMT) which was discovered 70 years ago methylates nicotinamide (NA) to generate 1-methyl nicotinamide. Its role in human health has evolved from serving only metabolic functions to being a driving force in a number of cancers. Although the increasing evidence showed NNMT was a feasible therapeutic target, its primary functions and mechanisms in cancer development, especially in tumor immunology, are not fully understood. The present study first thoroughly investigated the expression and prognostic significance of NNMT and the correlation between NNMT expression and immune cell infiltration in tumor microenvironment (TME) using bioinformatic techniques.

By analyzing the differences expression of NNMT in diverse tumor tissues and adjacent normal tissues, we found that the expression level of NNMT in the tumor tissues of BRCA, COAD, HNSC, KIRC, PAAD, and STAD is higher, while low NNMT expression was observed in bladder, liver, and lung cancers. The difference levels of NNMT expression in various cancer types may reflect distinct underlying mechanisms and functions. NNMT overexpression increases the chemoresistance through SIRT1 stabilization and activity in breast cancer [18]. NNMT depletion contributes to liver cancer cell survival by enhancing autophagy under nutrient starvation [10].

We further found that NNMT expression in different pathological stages of cancers was significantly different, consistent with the previous study showing that increased expression of NNMT was associated with increased tumor stage in STAD and OV [19, 20]. In fact, increased NNMT expression was associated with primary tumor size, lymph node metastasis, distant metastasis, and TNM stage in gastric cancer [19] and with increased tumor stage, grade, and mesenchymal molecular subtype in ovarian cancer [20] which indicated that NNMT might promote the growth and progression of cancer.

Upon analysis of the Kaplan-Meier plotter, upregulated expression of NNMT correlated with poor OS, RFS, or DSS in several cancers including bladder carcinoma, CESC, COAD, HNSC, KIRC, LUSC, LGG, OV, STAD, and THCA while correlated with good OS in sarcoma and UCEC.

Similar results also indicated that NNMT expression was significantly higher and correlated with poor survival in breast cancer [18], colorectal cancer [21], gastric cancer [19], and OV [20]. In regard to endometrial cancer, NNMT expression was significantly higher in primary high-grade and metastatic tumors and NNMT overexpression in metastatic tissue was associated with decreased survival [22]. These data conflict with our current results, possibly because only primary uterine corpus types of endometrial carcinoma are present in our study. Interestingly, NNMT was identified as a poor prognostic factor in CESC, HNSC, KIRC, LUSC, and LGG which has not been reported in previous studies. These results confirmed that NNMT was a potential biomarker for predicting the prognosis of cancer patients.

The emerging tumor microenvironment (TME) is a complex and continuously evolving entity. Early in tumor growth, a dynamic and reciprocal relationship develops between cancer cells and components of the TME to support cancer cell survival, local invasion, and metastatic dissemination [23]. Here, we found that NNMT expression presents a significantly positive correlation with both stromal and immune components of TME in pan-cancer.

Cancer-associated fibroblasts (CAFs) are important ingredients of the microenvironment in most types of cancers, and CAFs in the stroma of the TME have been reported to participate in modulating the function of various tumor-infiltrating immune cells [24, 25]. CAFs contribute to cancer immune escape through multiple mechanisms, such as secretion of multiple cytokines and chemokines, mediating the recruitment and functional differentiation of innate and adaptive immune cells [25]. Previous work has reported that NNMT is a central, metabolic regulator of CAF differentiation, and cancer progression in the stroma and inhibition of NNMT activity led to a reversion of the CAF phenotype [11]. High stromal NNMT is a prognostic marker in colorectal cancer [21], and NNMT enhances resistance to 5-fluorouracil in colorectal cancer cells through inhibition of the ASK1-p38 MAPK pathway [26]. NNMT induces cellular invasion via activating PI3K/Akt/SP1/MMP-2 pathway in clear cell renal cell carcinoma (ccRCC) [27]. The decreased activation of p44/42 MAPK and Akt following NNMT silencing shows a similar trend under in vivo conditions [28]. Plenty of evidence has confirmed that PI3K/AKT pathway promotes the differentiation of diverse cells into CAFs. PI3K/AKT signaling pathways regulated CAF-mediated cancer cell proliferation in many cancers including STAD and COAD [29]. MAPK signal was found to be involved not only in the metabolism of fatty acids but also in glycolysis in CAFs. Compared with these studies, our results showed that NNMT expression level had a positively correlations with infiltration levels of CAFs in 30 types of cancer. Our KEGG analysis suggested that NNMT was significantly associated with many cancer-promoting and immune-related pathway including PI3K-Akt, chemokine, cytokine in the immune system, cytokine-cytokine receptor interaction, cell adhesion, JAK-STAT, and MAPK signaling pathways. Combined with the previous research, our results serve as a reminder that NNMT regulates the CAFs in various cancers.

Immunoscore is a routine parameter in predicting the response to immunotherapy and should be considered as a prognostic factor for patients' survival [30]. Recently, with the breakthrough of immune checkpoints, immune checkpoint inhibitors, such as PD-1/PD-L1 inhibitors, have been widely used in the treatment of diverse cancers. The dynamic characteristics of the TME, tumor infiltrating cells, and immune biomarkers are important for the immunotherapy response [31]. Our current results showed that NNMT had a wider tumor applicability and was more closely related with immune cells in pan-cancer. NNMT significantly correlated with the infiltration levels of B cells, CD4^+^ T cells, CD8^+^ T cells, macrophages, NK cells, and neutrophils in diverse cancers. Specifically, NNMT expression had a strongly positive correlation with macrophages.

Macrophage levels were remarkably increased in the NNMT high expression group compared with that in the NNMT low expression group in COAD, HNSC, OV, and STAD. Among these cancer types, tumor-associated macrophage (TAM) markers such as CCL2, CD68, CD80, and IL10 had strong or moderate correlations with NNMT expression. M2 macrophage markers, for example, CD163, VSIG4, MRC1, and MS4A4A, also had strong or moderate correlations with NNMT expression while M1 macrophage markers, including INOS, IRF5, and ARG2, had negatively or no apparent correlations with NNMT expression. These findings indicated that NNMT may regulate the polarization of TAMs. TAMs in TME typically promote cancer cell proliferation, immunosuppression, and angiogenesis in support of tumor growth and metastasis. Oftentimes, the abundance of TAMs in tumor is correlated with poor disease prognosis. In colorectal cancer (CRC), TAMs induce EMT program to enhance CRC migration, invasion, and CTC-mediated metastasis by regulating the JAK2/STAT3/miR-506-3p/FoxQ1 axis, which in turn leads to the production of CCL2 that promote macrophage recruitment [32]. As for HNSC, HNSC cells drive TAMs towards M2 polarization. In turn, M2 TAMs contribute to migration and invasion of HNSC cells [33]. NNMT promotes epithelial-mesenchymal transition (EMT) in gastric cancer cells by activating transforming growth factor-*β*1 expression [34]. TAMs can also promote the invasiveness and migration properties of cancer cells by remodeling the extracellular matrix [35].

These above results indicate that NNMT may promote the differentiation of macrophages into TAMs involved in cancer progression resulting in poor prognosis. Together, our findings suggest that NNMT may play a key role in the regulation of cancer immune infiltrating, finally influencing prognosis of cancer patients.

## 5. Conclusions

In summary, we demonstrated that NNMT expression was related to clinicopathological characteristics and poor prognosis of pan-cancer. High expression of NNMT was closely related to CAF and immune cell infiltrations. High NNMT expression was predictive of macrophage infiltration and encouraged macrophage differentiation into TAMs in COAD, HNSC, OV, and STAD. These results displayed the prognostic value of NNMT and its potential role in tumor immunology.

Our study focused on the bioinformatic techniques of NNMT expression and patient survival utilizing multiple databases, without any verification experiments in vivo or in vitro. Future experimental studies of the NNMT expression and immune cell infiltration in different cancer populations may provide additional insights into the cancer mechanisms and the therapeutic strategies targeting NNMT to improve the therapeutic efficacy of immunotherapy.

## Figures and Tables

**Table 1:** Analysis of the correlation between NNMT and gene markers of TAMs, M1 macrophages, and M2 macrophages in TIMER.

Cell type	Gene markers	COAD				HNSC				OV				STAD			
		None		Purity		None		Purity		None		Purity		None		Purity	
		Cor	*P*	Cor	*P*	Cor	*P*	Cor	*P*	Cor	*P*	Cor	*P*	Cor	*P*	Cor	*P*
TAM	CCL2	0.73	∗∗∗∗	0.68	∗∗∗∗	0.37	∗∗∗∗	0.33	∗∗∗∗	0.48	∗∗∗∗	0.26	∗∗∗∗	0.59	∗∗∗∗	0.57	∗∗∗∗
	CD68	0.47	∗∗∗∗	0.40	∗∗∗∗	0.25	∗∗∗∗	0.19	∗∗∗∗	0.42	∗∗∗∗	0.14	∗	0.23	∗∗∗∗	0.19	∗∗∗∗
	CD80	0.51	∗∗∗∗	0.43	∗∗∗∗	0.37	∗∗∗∗	0.33	∗∗∗∗	0.36	∗∗∗∗	0.16	∗	0.27	∗∗∗∗	0.22	∗∗∗∗
	IL10	0.52	∗∗∗∗	0.46	∗∗∗∗	0.35	∗∗∗∗	0.30	∗∗∗∗	0.47	∗∗∗∗	0.27	∗∗∗∗	0.45	∗∗∗∗	0.42	∗∗∗∗

M1 macrophage	INOS (NOS2)	-0.14	∗∗∗	-0.21	∗∗∗∗	-0.17	∗∗∗∗	-0.13	∗∗∗	0.02	0.73	-0.06	0.37	-0.05	0.27	-0.08	0.13
	IRF5	0.26	∗∗∗∗	0.26	∗∗∗∗	-0.03	0.54	-0.04	0.441	0.11	0.06	-0.03	0.63	0.23	∗∗∗∗	0.24	∗∗∗∗
	ARG2	0.02	0.65	-0.02	0.75	-0.24	∗∗∗∗	-0.17	∗∗∗∗	0.18	∗∗	-0.14	∗	0.13	∗	-0.11	∗

M2 macrophage	CD163	0.67	∗∗∗∗	0.6	∗∗∗∗	0.47	∗∗∗∗	0.42	∗∗∗∗	0.43	∗∗∗∗	0.19	∗∗∗	0.42	∗∗∗∗	0.38	∗∗∗∗
	VSIG4	0.68	∗∗∗∗	0.61	∗∗∗∗	0.50	∗∗∗∗	0.45	∗∗∗∗	0.52	∗∗∗∗	0.292	∗∗∗∗	0.49	∗∗∗∗	0.47	∗∗∗∗
	MRC1	0.55	∗∗∗∗	0.47	∗∗∗∗	0.45	∗∗∗∗	0.38	∗∗∗∗	0.36	∗∗∗∗	0.11	0.08	0.38	∗∗∗∗	0.35	∗∗∗∗
	MS4A4A	0.64	∗∗∗∗	0.57	∗∗∗∗	0.50	∗∗∗∗	0.45	∗∗∗∗	0.49	∗∗∗∗	0.252	∗∗∗∗	0.46	∗∗∗∗	0.42	∗∗∗∗

None: correlation coefficient without adjustment; Purity: correlation adjusted by tumor purity; Cor: *R* value of Spearman's correlation. ^∗^*P* < 0.05. ^∗∗^*P* < 0.01. ^∗∗∗^*P* < 0.001. ^∗∗∗∗^*P* < 0.0001.

## Data Availability

The authors certify that all the original data in this research could be obtained from public database. NNMT gene expression in pan-cancer was verified in Oncomine (https://www.oncomine.org/), TIMER database (https://cistrome.shinyapps.io/timer), and UALCAN (http://ualcan.path.uab.edu/analysis). Survival data was verified in the Kaplan-Meier plotter (https://kmplot.com/analysis/) and PrognoScan (http://gepia.cancer-pku.cn/). Other data used to support the findings of this study are included within the supplementary information files. All the raw data of this study are available from the first author or corresponding author upon request.

## Supplementary Materials

Supplementary Materials Figure S1: correlation of NNMT gene expression with stromal score and immune score in different cancers. NNMT gene expression has a significantly positive correlation with the stromal score (A) and immune score (B) in COAD, HNSC, OV, and STAD. COAD: colon adenocarcinoma; HNSC: head and neck squamous cell carcinoma; NNMT: nicotinamide N-methyltransferase; OV: ovarian serous cystadenocarcinoma; STAD: stomach adenocarcinoma. Figure S2: macrophage infiltration in the NNMT high and low expression groups in COAD, HNSC, OV, and STAD. Compared with the NNMT low expression group, macrophages were significantly upregulated in the NNMT high expression group in COAD, HNSC, OV, and STAD (all *P* < 0.0001). COAD: colon adenocarcinoma; HNSC: head and neck squamous cell carcinoma; NNMT: nicotinamide N-methyltransferase; OV: ovarian serous cystadenocarcinoma; STAD: stomach adenocarcinoma. Table S1: the correlation of NNMT with stromal and immune scores. Table S2: the correlation of NNMT with immune cell infiltration in TCGA pan-cancer. Table S3: the association between NNMT expression and different immune cells in COAD, HNSC, OV, and STAD from ImmuCellAI database.
