# Supplementary material for: NNMT Is an Immune-Related Prognostic Biomarker That Modulates the Tumor Microenvironment in Pan-Cancer
Source: Dis Markers. 2023 Feb 9;2023:9226712. doi: 10.1155/2023/9226712 (PMC9934984; doi:10.1155/2023/9226712)
Supplement: Supplementary Materials — Figure S1: correlation of NNMT gene expression with stromal score and immune score in different cancers. NNMT gene expression has a significantly positive correlation with the stromal score (A) and immune score (B) in COAD, HNSC, OV, and STAD. COAD: colon adenocarcinoma; HNSC: head and neck squamous cell carcinoma; NNMT: nicotinamide N-methyltransferase; OV: ovarian serous cystadenocarcinoma; STAD: stomach adenocarcinoma. Figure S2: macrophage infiltration in the NNMT high and low expression groups in COAD, HNSC, OV, and STAD. Compared with the NNMT low expression group, macrophages were significantly upregulated in the NNMT high expression group in COAD, HNSC, OV, and STAD (all P < 0.0001). COAD: colon adenocarcinoma; HNSC: head and neck squamous cell carcinoma; NNMT: nicotinamide N-methyltransferase; OV: ovarian serous cystadenocarcinoma; STAD: stomach adenocarcinoma. Table S1: the correlation of NNMT with stromal and immune scores. Table S2: the correlation of NNMT with immune cell infiltration in TCGA pan-cancer. Table S3: the association between NNMT expression and different immune cells in COAD, HNSC, OV, and STAD from ImmuCellAI database. [file 9226712.f1.zip › Table S2.docx]

**Supplementary Table2. The correlation of NNMT with immune cell infiltration in TCGA pan-cancer**

| **The correlation of NNMT with B cells infiltration in TCGA pan-cancer** | | | |
| --- | --- | --- | --- |
| **cancer** | **infiltrates** | **correlation** | ***P* value** |
| THCA | B_cell | 0.226439685 | 2.49E-07 |
| COAD | B_cell | 0.262976667 | 6.09E-06 |
| GBM | B_cell | -0.27689584 | 0.000345988 |
| UVM | B_cell | 0.378745456 | 0.000577685 |
| THYM | B_cell | 0.304570429 | 0.000757747 |
| LUSC | B_cell | 0.149770395 | 0.000800003 |
| PRAD | B_cell | 0.148652732 | 0.0008977 |
| HNSC | B_cell | -0.10787019 | 0.013851789 |
| SKCM | B_cell | 0.106326901 | 0.021276254 |
| PCPG | B_cell | 0.15869527 | 0.032375464 |
| LGG | B_cell | -0.087335551 | 0.046107056 |
| KIRP | B_cell | 0.110020784 | 0.061772979 |
| PAAD | B_cell | 0.137988788 | 0.065467508 |
| ESCA | B_cell | 0.136580488 | 0.065988561 |
| BLCA | B_cell | 0.067147016 | 0.176375308 |
| KICH | B_cell | -0.142594553 | 0.253388509 |
| STAD | B_cell | 0.049580027 | 0.314824802 |
| DLBC | B_cell | -0.147445165 | 0.322647004 |
| TGCT | B_cell | 0.068549312 | 0.426070189 |
| LIHC | B_cell | -0.034285236 | 0.510316206 |
| MESO | B_cell | -0.068132575 | 0.530635674 |
| BRCA | B_cell | 0.018545031 | 0.539491396 |
| LUAD | B_cell | 0.022262668 | 0.614568377 |
| ACC | B_cell | -0.057554392 | 0.619055343 |
| READ | B_cell | 0.042127638 | 0.690094788 |
| CESC | B_cell | 0.012627548 | 0.82587379 |
| UCS | B_cell | 0.026950839 | 0.842260735 |
| SARC | B_cell | 0.009298965 | 0.880922064 |
| OV | B_cell | -0.006703199 | 0.907187375 |
| UCEC | B_cell | -0.00803294 | 0.914531036 |
| CHOL | B_cell | 0.010067879 | 0.953528158 |
| KIRC | B_cell | -0.000533254 | 0.990218941 |
| **The correlation of NNMT with CD4^+^ T cells infiltration in TCGA pan-cancer** | | | |
| **cancer** | **infiltrates** | **correlation** | ***P* value** |
| LGG | T_cell_CD4 | 0.464430757 | 2.75E-29 |
| PRAD | T_cell_CD4 | 0.416075784 | 3.47E-22 |
| THCA | T_cell_CD4 | 0.385529014 | 1.90E-19 |
| COAD | T_cell_CD4 | 0.47329678 | 1.75E-17 |
| BLCA | T_cell_CD4 | 0.348111729 | 4.88E-13 |
| BRCA | T_cell_CD4 | 0.19889849 | 3.00E-11 |
| LUSC | T_cell_CD4 | 0.263963695 | 2.20E-09 |
| PCPG | T_cell_CD4 | 0.369261029 | 2.90E-07 |
| KIRP | T_cell_CD4 | 0.286249238 | 7.46E-07 |
| KICH | T_cell_CD4 | 0.548620996 | 1.85E-06 |
| ESCA | T_cell_CD4 | 0.296788035 | 4.73E-05 |
| SKCM | T_cell_CD4 | 0.185962166 | 5.08E-05 |
| TGCT | T_cell_CD4 | 0.335733111 | 6.05E-05 |
| STAD | T_cell_CD4 | 0.195699762 | 6.23E-05 |
| UCEC | T_cell_CD4 | 0.288135377 | 8.38E-05 |
| SARC | T_cell_CD4 | 0.212874464 | 0.000522313 |
| READ | T_cell_CD4 | 0.320335579 | 0.001851909 |
| THYM | T_cell_CD4 | -0.276512873 | 0.002333431 |
| PAAD | T_cell_CD4 | 0.208637591 | 0.005067105 |
| OV | T_cell_CD4 | 0.146436367 | 0.010445591 |
| KIRC | T_cell_CD4 | 0.108025786 | 0.012748756 |
| UVM | T_cell_CD4 | -0.224785208 | 0.046408781 |
| LUAD | T_cell_CD4 | -0.079005963 | 0.073513986 |
| ACC | T_cell_CD4 | 0.185703366 | 0.105881313 |
| CESC | T_cell_CD4 | 0.065125369 | 0.256051865 |
| GBM | T_cell_CD4 | 0.087203808 | 0.268343403 |
| DLBC | T_cell_CD4 | -0.134473391 | 0.367492038 |
| UCS | T_cell_CD4 | 0.109243674 | 0.418558667 |
| MESO | T_cell_CD4 | -0.081087419 | 0.455290301 |
| HNSC | T_cell_CD4 | 0.021103584 | 0.631134157 |
| LIHC | T_cell_CD4 | 0.016525714 | 0.751049919 |
| CHOL | T_cell_CD4 | -0.000173645 | 0.999198045 |
| **The correlation of NNMT with CD8^+^ T cells infiltration in TCGA pan-cancer** | | | |
| **cancer** | **infiltrates** | **correlation** | ***P* value** |
| LGG | T_cell_CD8 | 0.376214987 | 5.38E-19 |
| THCA | T_cell_CD8 | 0.338570159 | 4.33E-15 |
| BLCA | T_cell_CD8 | 0.371245966 | 9.55E-15 |
| PRAD | T_cell_CD8 | 0.30231625 | 6.09E-12 |
| ACC | T_cell_CD8 | 0.583400152 | 2.58E-08 |
| COAD | T_cell_CD8 | 0.31062698 | 7.35E-08 |
| STAD | T_cell_CD8 | 0.259775824 | 8.53E-08 |
| KIRP | T_cell_CD8 | 0.275343842 | 2.01E-06 |
| KICH | T_cell_CD8 | 0.544358468 | 2.31E-06 |
| LUSC | T_cell_CD8 | 0.197272959 | 9.21E-06 |
| PCPG | T_cell_CD8 | 0.314738595 | 1.51E-05 |
| UVM | T_cell_CD8 | 0.455491932 | 2.47E-05 |
| BRCA | T_cell_CD8 | 0.126476172 | 2.66E-05 |
| PAAD | T_cell_CD8 | 0.271657432 | 0.000234622 |
| SKCM | T_cell_CD8 | 0.161914327 | 0.000430927 |
| READ | T_cell_CD8 | 0.339500081 | 0.000930432 |
| OV | T_cell_CD8 | 0.188514564 | 0.000938108 |
| ESCA | T_cell_CD8 | 0.236994067 | 0.001276496 |
| SARC | T_cell_CD8 | 0.174445521 | 0.004627347 |
| TGCT | T_cell_CD8 | 0.232020291 | 0.006368521 |
| UCEC | T_cell_CD8 | 0.201000709 | 0.006663684 |
| GBM | T_cell_CD8 | 0.208062453 | 0.007695354 |
| THYM | T_cell_CD8 | 0.240081522 | 0.008540561 |
| KIRC | T_cell_CD8 | 0.092365102 | 0.03334171 |
| LIHC | T_cell_CD8 | 0.108127364 | 0.03736448 |
| DLBC | T_cell_CD8 | -0.303675475 | 0.037975465 |
| UCS | T_cell_CD8 | 0.230483304 | 0.084551513 |
| CHOL | T_cell_CD8 | 0.246181154 | 0.147801457 |
| LUAD | T_cell_CD8 | -0.035306202 | 0.424437001 |
| CESC | T_cell_CD8 | 0.032349577 | 0.572946024 |
| HNSC | T_cell_CD8 | -0.01423057 | 0.746133077 |
| MESO | T_cell_CD8 | -0.008783543 | 0.935645543 |
| **The correlation of NNMT with macrophages infiltration in TCGA pan-cancer** | | | |
| **cancer** | **infiltrates** | **correlation** | ***P* value** |
| BLCA | Macrophage | 0.55744695 | 1.32E-34 |
| LGG | Macrophage | 0.487376786 | 1.70E-32 |
| LUSC | Macrophage | 0.408193985 | 2.03E-21 |
| LIHC | Macrophage | 0.441826591 | 3.69E-19 |
| HNSC | Macrophage | 0.355976711 | 5.58E-17 |
| COAD | Macrophage | 0.458480041 | 2.24E-16 |
| SARC | Macrophage | 0.433401888 | 2.01E-13 |
| OV | Macrophage | 0.398227956 | 4.93E-13 |
| PRAD | Macrophage | 0.315926983 | 5.86E-13 |
| READ | Macrophage | 0.625676967 | 2.59E-11 |
| SKCM | Macrophage | 0.295364292 | 6.77E-11 |
| PCPG | Macrophage | 0.451319023 | 1.61E-10 |
| GBM | Macrophage | 0.425293411 | 1.52E-08 |
| UVM | Macrophage | 0.576889742 | 2.63E-08 |
| ESCA | Macrophage | 0.391787891 | 4.52E-08 |
| THCA | Macrophage | 0.225091981 | 2.95E-07 |
| STAD | Macrophage | 0.226142326 | 3.45E-06 |
| TGCT | Macrophage | 0.38349947 | 3.73E-06 |
| BRCA | Macrophage | 0.135435684 | 6.75E-06 |
| KICH | Macrophage | 0.50780801 | 1.35E-05 |
| KIRP | Macrophage | 0.242828939 | 3.01E-05 |
| UCEC | Macrophage | 0.250033441 | 0.000687208 |
| UCS | Macrophage | 0.425228754 | 0.000976194 |
| ACC | Macrophage | 0.365231019 | 0.001090464 |
| THYM | Macrophage | 0.275376409 | 0.002436444 |
| CHOL | Macrophage | 0.488926574 | 0.002479209 |
| DLBC | Macrophage | 0.410124166 | 0.004195296 |
| PAAD | Macrophage | 0.196689197 | 0.008317179 |
| MESO | Macrophage | 0.144297118 | 0.182388327 |
| CESC | Macrophage | 0.060154264 | 0.294217968 |
| LUAD | Macrophage | 0.022321979 | 0.613625515 |
| KIRC | Macrophage | 0.010035491 | 0.817537737 |
| **The correlation of NNMT with neutrophils infiltration in TCGA pan-cancer** | | | |
| **cancer** | **infiltrates** | **correlation** | ***P* value** |
| LGG | Neutrophil | 0.513757458 | 1.69E-36 |
| THCA | Neutrophil | 0.504139002 | 4.21E-34 |
| BLCA | Neutrophil | 0.489814566 | 6.01E-26 |
| LUSC | Neutrophil | 0.445758329 | 1.10E-25 |
| COAD | Neutrophil | 0.557511912 | 6.39E-25 |
| BRCA | Neutrophil | 0.276032383 | 1.24E-20 |
| PRAD | Neutrophil | 0.383905795 | 7.28E-19 |
| STAD | Neutrophil | 0.389240803 | 2.17E-16 |
| SKCM | Neutrophil | 0.341199191 | 2.99E-14 |
| OV | Neutrophil | 0.403595558 | 2.24E-13 |
| KIRP | Neutrophil | 0.403502681 | 9.64E-13 |
| PCPG | Neutrophil | 0.489655666 | 2.30E-12 |
| PAAD | Neutrophil | 0.453828136 | 1.77E-10 |
| HNSC | Neutrophil | 0.268810149 | 4.66E-10 |
| GBM | Neutrophil | 0.386175003 | 3.56E-07 |
| ACC | Neutrophil | 0.533754359 | 5.77E-07 |
| KIRC | Neutrophil | 0.211600864 | 8.64E-07 |
| UCEC | Neutrophil | 0.345020721 | 1.97E-06 |
| ESCA | Neutrophil | 0.339391545 | 2.77E-06 |
| SARC | Neutrophil | 0.279566428 | 4.32E-06 |
| READ | Neutrophil | 0.430900597 | 1.80E-05 |
| LIHC | Neutrophil | 0.188369079 | 0.000263553 |
| KICH | Neutrophil | 0.341169644 | 0.005057332 |
| TGCT | Neutrophil | 0.183269801 | 0.032060621 |
| UVM | Neutrophil | -0.229591145 | 0.041810598 |
| UCS | Neutrophil | 0.237595546 | 0.075133837 |
| MESO | Neutrophil | 0.187450574 | 0.082108545 |
| CESC | Neutrophil | 0.099500061 | 0.082258149 |
| DLBC | Neutrophil | -0.151182157 | 0.310397238 |
| CHOL | Neutrophil | 0.05669732 | 0.742577048 |
| THYM | Neutrophil | -0.018845454 | 0.838800726 |
| LUAD | Neutrophil | -0.003896182 | 0.92978302 |
| **The correlation of NNMT with natural killer (NK) cells infiltration in TCGA pan-cancer** | | | |
| **cancer** | **infiltrates** | **correlation** | ***P* value** |
| HNSC | NK_cell | 0.277445436 | 1.21E-10 |
| BLCA | NK_cell | 0.274448273 | 1.82E-08 |
| ACC | NK_cell | 0.520215902 | 1.24E-06 |
| STAD | NK_cell | 0.178963745 | 0.000256677 |
| CESC | NK_cell | 0.203786635 | 0.000333297 |
| THCA | NK_cell | 0.154610123 | 0.000470167 |
| COAD | NK_cell | 0.203608989 | 0.000507394 |
| PCPG | NK_cell | 0.243774474 | 0.000912468 |
| BRCA | NK_cell | 0.097438499 | 0.001232407 |
| KIRP | NK_cell | 0.168277565 | 0.004120244 |
| MESO | NK_cell | 0.299557916 | 0.004821639 |
| ESCA | NK_cell | 0.204382418 | 0.005647981 |
| LUSC | NK_cell | 0.115593933 | 0.009829748 |
| TGCT | NK_cell | 0.21260821 | 0.012620604 |
| KIRC | NK_cell | 0.106707731 | 0.013888056 |
| LGG | NK_cell | 0.107534062 | 0.013967169 |
| THYM | NK_cell | 0.199171474 | 0.029887591 |
| OV | NK_cell | 0.12112474 | 0.034476187 |
| PRAD | NK_cell | 0.094213892 | 0.035939028 |
| GBM | NK_cell | 0.164210404 | 0.036206216 |
| UCEC | NK_cell | 0.129992841 | 0.081130016 |
| SKCM | NK_cell | 0.072468209 | 0.117050585 |
| SARC | NK_cell | 0.090971046 | 0.141968226 |
| PAAD | NK_cell | 0.090364301 | 0.228982192 |
| UVM | NK_cell | 0.127271012 | 0.263681746 |
| READ | NK_cell | 0.106675074 | 0.311493575 |
| DLBC | NK_cell | 0.102927562 | 0.491158967 |
| CHOL | NK_cell | -0.098167664 | 0.568950626 |
| LUAD | NK_cell | 0.020768877 | 0.638521284 |
| UCS | NK_cell | 0.060391473 | 0.655415203 |
| LIHC | NK_cell | 0.011846591 | 0.820097605 |
| KICH | NK_cell | 0.018064441 | 0.885528979 |
| **The correlation of NNMT with Cancer-associated fibroblasts (CAFs) infiltration in TCGA pan-cancer** | | | |
| **cancer** | **infiltrates** | **correlation** | ***P* value** |
| BRCA | CAFs | 0.567292492 | 1.94E-94 |
| STAD | CAFs | 0.606971536 | 6.29E-43 |
| LUSC | CAFs | 0.560435137 | 1.58E-42 |
| BLCA | CAFs | 0.600325406 | 3.28E-41 |
| COAD | CAFs | 0.682384105 | 8.26E-41 |
| THCA | CAFs | 0.528381121 | 7.14E-38 |
| HNSC | CAFs | 0.496640159 | 9.73E-34 |
| PRAD | CAFs | 0.443791313 | 2.37E-25 |
| LGG | CAFs | 0.419453795 | 1.16E-23 |
| PCPG | CAFs | 0.565279824 | 9.30E-17 |
| OV | CAFs | 0.443803012 | 3.76E-16 |
| ESCA | CAFs | 0.538048628 | 4.77E-15 |
| PAAD | CAFs | 0.496957413 | 1.49E-12 |
| READ | CAFs | 0.653934533 | 1.58E-12 |
| SKCM | CAFs | 0.299815978 | 3.39E-11 |
| CESC | CAFs | 0.299097243 | 9.65E-08 |
| LUAD | CAFs | 0.213336851 | 1.05E-06 |
| ACC | CAFs | 0.512621488 | 1.88E-06 |
| UCEC | CAFs | 0.343929881 | 2.13E-06 |
| UVM | CAFs | 0.487165694 | 5.29E-06 |
| KIRP | CAFs | 0.256185026 | 1.03E-05 |
| KICH | CAFs | 0.460010577 | 0.000101925 |
| SARC | CAFs | 0.23036118 | 0.000168825 |
| DLBC | CAFs | 0.502641158 | 0.000317606 |
| GBM | CAFs | 0.269685527 | 0.000498898 |
| THYM | CAFs | 0.311090512 | 0.000573949 |
| LIHC | CAFs | 0.162489033 | 0.001688949 |
| UCS | CAFs | 0.355299243 | 0.006685177 |
| KIRC | CAFs | 0.107024203 | 0.013606621 |
| TGCT | CAFs | 0.186602261 | 0.029011832 |
| CHOL | CAFs | 0.257008864 | 0.130234132 |
| MESO | CAFs | 0.03029201 | 0.780611428 |
| **The correlation of NNMT with endothelial cells infiltration in TCGA pan-cancer** | | | |
| **cancer** | **infiltrates** | **correlation** | ***P* value** |
| BRCA | Endothelial | 0.370645325 | 4.70E-37 |
| LUSC | Endothelial | 0.493077537 | 7.18E-32 |
| COAD | Endothelial | 0.554268618 | 1.36E-24 |
| STAD | Endothelial | 0.436597543 | 1.19E-20 |
| HNSC | Endothelial | 0.38648744 | 5.69E-20 |
| PCPG | Endothelial | 0.536327871 | 6.04E-15 |
| PRAD | Endothelial | 0.338800411 | 8.70E-15 |
| BLCA | Endothelial | 0.313369956 | 1.01E-10 |
| SKCM | Endothelial | 0.247868069 | 5.37E-08 |
| THCA | Endothelial | -0.234047886 | 9.48E-08 |
| ESCA | Endothelial | 0.335186143 | 3.74E-06 |
| CESC | Endothelial | 0.246707017 | 1.27E-05 |
| TGCT | Endothelial | 0.361647855 | 1.41E-05 |
| KIRC | Endothelial | 0.184176235 | 1.95E-05 |
| THYM | Endothelial | 0.374573304 | 2.71E-05 |
| LGG | Endothelial | 0.181170901 | 3.13E-05 |
| READ | Endothelial | 0.403646391 | 6.61E-05 |
| UCEC | Endothelial | 0.242644304 | 0.000997774 |
| PAAD | Endothelial | 0.236366096 | 0.001445196 |
| LUAD | Endothelial | 0.139679382 | 0.001500345 |
| ACC | Endothelial | 0.344687441 | 0.002141942 |
| LIHC | Endothelial | 0.145641904 | 0.004941898 |
| SARC | Endothelial | 0.172453949 | 0.005125076 |
| UCS | Endothelial | 0.340626181 | 0.009521561 |
| KIRP | Endothelial | 0.138245826 | 0.018708215 |
| DLBC | Endothelial | 0.302039425 | 0.039076079 |
| UVM | Endothelial | 0.22196495 | 0.049296182 |
| OV | Endothelial | 0.07488535 | 0.192138672 |
| MESO | Endothelial | 0.075929609 | 0.484557832 |
| KICH | Endothelial | 0.085703941 | 0.493836639 |
| GBM | Endothelial | 0.005721322 | 0.942217223 |
| CHOL | Endothelial | 0.011956106 | 0.944824614 |
